# Efficacy of Real-Time Continuous Glucose Monitoring in Improving Glycemic Outcomes Among Adults With Type 2 Diabetes: A Systematic Review of Randomized Controlled Trials

**DOI:** 10.7759/cureus.89534

**Published:** 2025-08-07

**Authors:** Shaza Awad Ali Badawi, Ali Hadi M Alhajri, Nisreen Mohamed Osman Altayb, Enas Mohmmed, Rayan Kamil Elamin Hassabelrasoul, Shahd Abdullahi Sidahmed Mohammed, Wamda Hussein Altahir Mohammed, Jihad A El Ghali

**Affiliations:** 1 Department of Internal Medicine, Yanbu General Hospital, Madinah, SAU; 2 Endocrinology, Najran Armed Forces Hospital, Ministry of Defense Health Services, Najran, SAU; 3 Endocrinology, Nottingham University Hospital, Nottingham, GBR; 4 General Medicine, Tallaght University Hospital, Dublin, IRL; 5 Internal Medicine, Al-Sulayyil General Hospital, As Sulayyil, SAU; 6 Internal Medicine, Jaber Al-Ahmad Al-Sabah Hospital, Hawally, KWT; 7 Internal Medicine, Al Duqm Hospital, Al Duqm, OMN; 8 Internal Medicine, Dr. Samir Abbas Hospital, Jeddah, SAU

**Keywords:** glycemic control, hba1c, real-time continuous glucose monitoring, rt-cgm, systematic review, time in range, type 2 diabetes

## Abstract

Type 2 diabetes (T2D) requires rigorous glycemic control to prevent complications, but traditional self-monitoring of blood glucose (SMBG) offers limited insights. Real-time continuous glucose monitoring (RT-CGM) provides dynamic data to optimize management, although its efficacy in T2D remains debated. This systematic review synthesizes evidence from randomized controlled trials (RCTs) to evaluate RT-CGM’s impact on glycemic outcomes in adults with T2D. Following Preferred Reporting Items for Systematic reviews and Meta-Analyses (PRISMA) 2020 guidelines, we searched PubMed, Scopus, Web of Science, and ClinicalTrials.gov (2015-2025) for RCTs comparing RT-CGM to SMBG/usual care in non-pregnant adults with T2D. Eleven studies met the inclusion criteria. Data were extracted for HbA1c, time in range (TIR), hypoglycemia, and safety. Risk of bias was assessed using Cochrane RoB 2. RT-CGM significantly improved TIR and reduced HbA1c in insulin-treated patients, although benefits varied by intervention frequency and patient subgroup. Episodic use required multiple sessions for sustained HbA1c reduction. Non-insulin-treated cohorts saw smaller HbA1c changes but improved glycemic variability. Treatment satisfaction was consistently higher with RT-CGM. Safety profiles were favorable, with no severe device-related adverse events. RT-CGM enhances glycemic control in T2D, particularly for insulin-treated patients, with structured use yielding the greatest benefits. Clinicians should prioritize individualized protocols and patient education. Future research should address long-term efficacy and cost-effectiveness.

## Introduction and background

Type 2 diabetes mellitus (T2DM) is a chronic metabolic disorder characterized by insulin resistance and impaired insulin secretion, leading to persistent hyperglycemia [1]. Effective glycemic control is crucial for preventing the progression of diabetes-related complications such as retinopathy, nephropathy, neuropathy, and cardiovascular diseases [2]. Traditionally, self-monitoring of blood glucose (SMBG) using capillary blood samples has been the mainstay for assessing glycemic status and guiding therapy adjustments [3]. However, SMBG provides only intermittent snapshots of blood glucose levels, potentially missing important fluctuations such as postprandial hyperglycemia or nocturnal hypoglycemia [4].

Real-time continuous glucose monitoring (RT-CGM) systems have emerged as an innovative technology that offers dynamic and continuous assessment of interstitial glucose levels throughout the day and night [5]. Unlike SMBG, RT-CGM provides immediate feedback on glucose trends and alerts for impending hypo- or hyperglycemia, enabling timely therapeutic and behavioral interventions [6]. Several randomized controlled trials (RCTs) have evaluated the impact of RT-CGM on glycemic outcomes among individuals with T2DM, with varying results across different study populations and durations of follow-up.

Despite the increasing adoption of RT-CGM in clinical practice, the overall efficacy of RT-CGM compared to conventional SMBG in improving glycemic outcomes such as HbA1c reduction, time in range (TIR), and hypoglycemia prevention among adults with T2DM remains to be systematically synthesized. Therefore, this systematic review aims to comprehensively evaluate the efficacy of RT-CGM in improving glycemic outcomes among adults with T2DM based on evidence from RCTs. The findings from this review will help inform clinical decision-making and guideline development for optimizing diabetes management through technology-assisted glucose monitoring.

## Review

Methodology

Review Protocol

This systematic review was conducted in accordance with the Preferred Reporting Items for Systematic Reviews and Meta-Analyses (PRISMA) 2020 guidelines to ensure methodological rigor and transparency [7].

Eligibility Criteria

Studies were eligible for inclusion if they were RCTs evaluating the efficacy of RT-CGM in improving glycemic outcomes among adults (aged 18 years and above) diagnosed with T2DM. Eligible studies had to report at least one glycemic outcome, such as changes in HbA1c, TIR, time above range (TAR), time below range (TBR), or incidence of hypoglycemia. Only articles published in English between January 2015 and July 2025 were considered to ensure the inclusion of research reflecting recent advancements in RT-CGM technology and contemporary clinical practices. Studies were excluded if they involved participants with type 1 diabetes mellitus (T1DM), gestational diabetes, or other specific types of diabetes; included pediatric populations or pregnant women; or utilized blinded CGM devices that did not provide real-time glucose data to participants. In addition, non-randomized studies, observational research, reviews, conference abstracts, editorials, and study protocols were excluded.

Information Sources and Search Strategy

A comprehensive literature search was conducted across four major electronic databases: PubMed, Scopus, Web of Science, and ClinicalTrials.gov. The search strategy combined keywords and Medical Subject Headings (MeSH) related to “real-time continuous glucose monitoring", “RT-CGM”, “type 2 diabetes mellitus”, and “randomized controlled trials”. Boolean operators were used to refine and expand search results where appropriate. In addition, reference lists of included studies and relevant reviews were manually screened to identify any additional eligible studies. The search was restricted to the last 10 years (2015-2025) to include only recent literature, as RT-CGM technologies have evolved significantly in the past decade, with improved accuracy, usability, and integration with digital health platforms.

Study Selection

All identified records were imported into EndNote reference management software, and duplicates were removed. Two reviewers independently screened titles and abstracts for relevance, followed by full-text screening against the inclusion criteria. Discrepancies were resolved through discussion or consultation with a third reviewer to ensure objectivity and consensus in study selection.

Data Extraction

A standardized data extraction form was developed and pilot tested. Data were extracted independently by two reviewers and cross-checked for accuracy. Extracted information included first author, year of publication, country, study design, sample size, population characteristics, intervention details (RT-CGM specifications and usage protocols), comparator details (e.g., SMBG frequency), duration of follow-up, primary glycemic outcomes measured (such as HbA1c, time in range, hypoglycemia events), and key findings.

Risk-of-Bias Assessment

The risk of bias for included studies was assessed independently by two reviewers using the Cochrane Risk of Bias 2 (RoB 2) tool for RCTs [8]. This tool evaluates bias across five domains: the randomization process, deviations from intended interventions, missing outcome data, measurement of outcomes, and selection of the reported result. Any disagreements in judgments were resolved through discussion to reach a consensus.

Data Synthesis

Due to substantial heterogeneity in the included studies in terms of intervention protocols, duration of follow-up, outcome measures, and reporting methods, a meta-analysis was not conducted. The diversity of RT-CGM devices used, varying baseline HbA1c levels of participants, differences in comparator groups (e.g., frequency and type of SMBG), and the diversity of reported glycemic outcomes would have rendered pooled estimates unreliable and potentially misleading. Therefore, findings from the included studies were synthesized narratively, highlighting study characteristics, intervention details, and key outcomes.

Reporting

The systematic review findings are reported in line with the PRISMA 2020 checklist to ensure transparency, replicability, and completeness. A PRISMA flow diagram is included to illustrate the process of study selection from initial screening to final inclusion.

Results

Search Results

**Figure 1:**
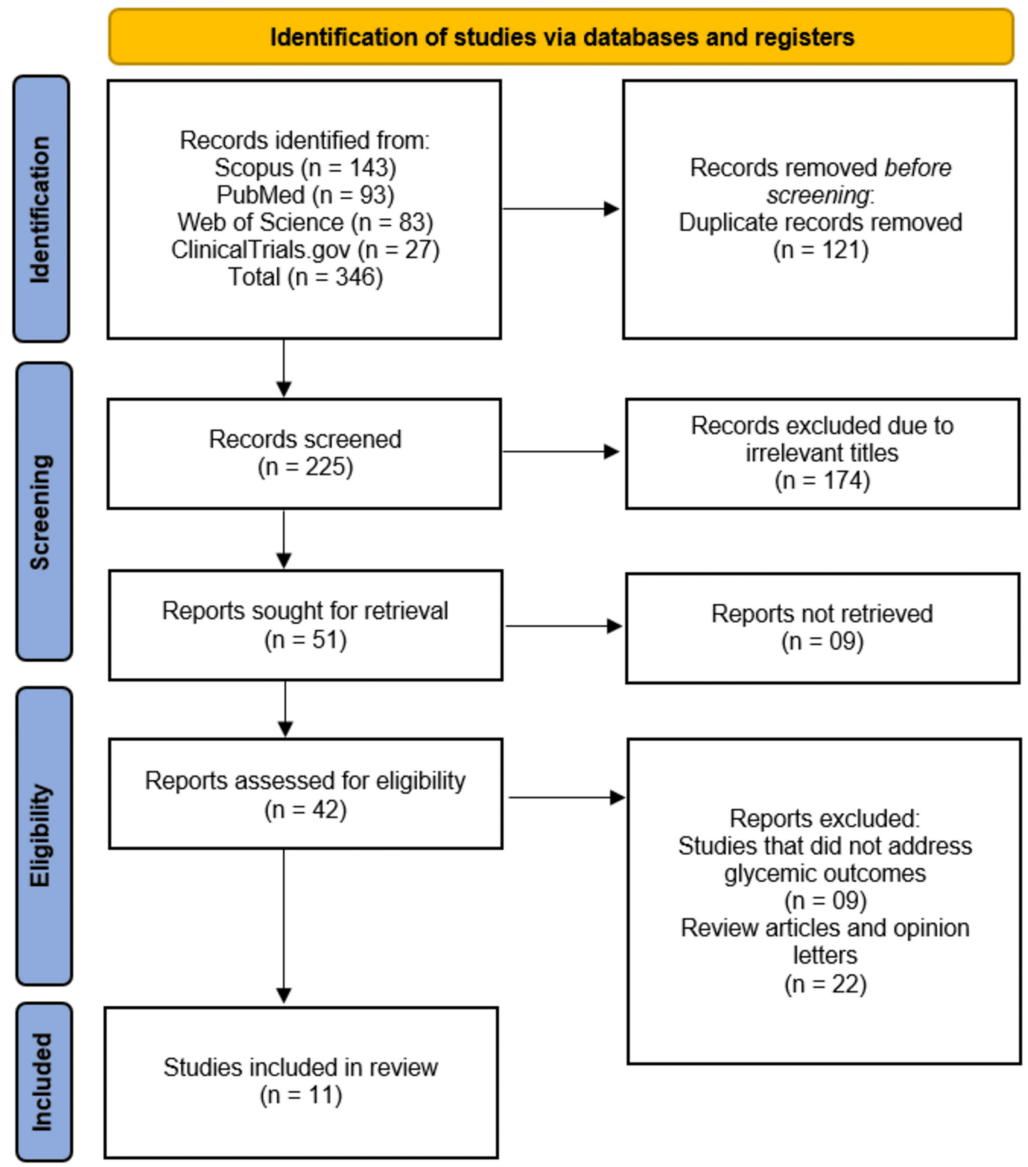
Preferred Reporting Items for Systematic Reviews and Meta-Analyses (PRISMA) flowchart of the study selection process

The systematic search was conducted across four databases (Scopus, PubMed, Web of Science, and ClinicalTrials.gov), yielding 346 records after duplicate removal. Initial screening of 225 records excluded 174 studies based on irrelevant titles. Of the remaining 51 reports, nine were unavailable for retrieval, and 31 were excluded during eligibility assessment (nine for lacking glycemic outcomes and 22 for being review articles/opinion letters). Ultimately, 11 RCTs [9-19] met the inclusion criteria and were selected for this systematic review (Figure 1).

Study Characteristics

The systematic review included 11 RCTs [9-19] published between 2016 and 2023, evaluating the efficacy of RT-CGM in adults with T2D. The studies varied in design, sample size, intervention protocols, and follow-up durations, as summarized in Table 1. Populations included non-insulin-treated and insulin-treated T2D patients, with baseline HbA1c levels ranging from 7.8% to 10.5%. Interventions included episodic RT-CGM use, flash glucose monitoring (FGM), and professional CGM systems, with comparators primarily being SMBG or usual care. Follow-up periods ranged from 12 weeks to 12 months, with glycemic outcomes measured as changes in HbA1c, TIR, and other secondary metrics [9-11].

**Table 1:** Characteristics of the included studies RT-CGM: real-time continuous glucose monitoring, SMBG: self-monitoring of blood glucose, HbA1c; hemoglobin A1c (glycated hemoglobin), TIR; time in range, CGM: continuous glucose monitoring, FGM: flash glucose monitoring, RCT: randomized controlled trial, SD: standard deviation, T2D: type 2 diabetes, T2DM: type 2 diabetes mellitus, BGM: blood glucose monitoring, QoL: quality of life, GP-OSMOTIC: General Practice Optimising Structured Monitoring to Improve Clinical Outcomes Trial, IQR: interquartile range, DM: diabetes mellitus

Author (year)	Country / setting	Study design	Sample size (n)	Population characteristics
Price et al. [9] (2021)	North America (eight sites)	Randomized pilot trial	68 (45 rtCGM; 23 SMBG)	Adults with T2D using ≥2 non-insulin therapies, HbA1c 7.8–10.5%
Moon et al. [10] (2023)	South Korea	Randomized prospective study	61 (48 completed)	Non-insulin–treated T2D patients uncontrolled with oral antidiabetic drugs (baseline HbA1c 8.2% ± 0.5%)
Wada et al. [11] (2020)	Japan / five hospitals	Multicenter, open-label, randomized (1:1), parallel-group RCT	Total n = 100 (FGM: 49, SMBG: 51)	Adults with non-insulin-treated type 2 diabetes
Ajjan et al. [12] (2019)	UK (Primary and secondary care centres)	Randomized controlled trial	148	Adults with type 2 diabetes on insulin therapy
Yeoh et al. [13] (2018)	Singapore	Randomized controlled trial (pilot)	30 (CGM: 14, SMBG: 16)	Adults with type 2 diabetes and stage 3 diabetic kidney disease, HbA1c >8%, majority >10 years diabetes duration, ~90% on insulin therapy
Sato et al. [14] (2016)	Japan	Randomized controlled trial	34	Adults with poorly controlled T2DM treated with insulin; baseline HbA1c ~8.2%
Martens et al. [15] (2021)	United States / 15 primary care centers	Randomized clinical trial	175 (CGM: 116; BGM: 59)	Adults with type 2 diabetes treated with basal insulin only (mean age 57 years; 50% female; 53% racial/ethnic minorities; mean baseline HbA1c 9.1%)
Haak et al. [16] (2017)	26 European diabetes centers	Open-label randomized controlled trial	224 (149 intervention, 75 control)	Adults with type 2 diabetes on intensive insulin therapy
Furler et al. [17] (2020)	Australia / 25 general practices in Victoria	Pragmatic, two-arm, open-label, individually randomized controlled trial (GP-OSMOTIC)	299 (149 intervention, 150 control)	Adults aged 18–80 years with type 2 diabetes for ≥1 year, HbA1c ≥0.5% above target despite ≥2 non-insulin glucose-lowering drugs or insulin, therapy stable ≥4 months
Beck et al. [18] (2017)	North America (25 endocrinology practices)	Randomized clinical trial	158 (CGM: 79, control: 79)	Adults with type 2 diabetes, median diabetes duration 17 years (IQR 11–23), age 35–79 years (mean 60 ±10), HbA1c 7.5–9.9% (mean 8.5%), receiving multiple daily insulin injections
Ajjan et al. [19] (2016)	UK (multicenter study)	Prospective randomized controlled trial	87 (intervention: 59, control: 28)	Insulin-treated patients with type 1 and type 2 diabetes

Glycemic Outcomes

The primary outcome of interest was HbA1c reduction, which demonstrated mixed results across studies. Martens et al. [15] reported a significant between-group difference in HbA1c reduction (−0.4%, 95% CI −0.8% to −0.1%; p = 0.02) favoring RT-CGM over traditional BGM in basal insulin-treated patients. Similarly, Beck et al. [18] observed a modest but statistically significant HbA1c reduction (−0.3%, 95% CI −0.5% to 0.0%; p = 0.022) in patients using RT-CGM compared to usual care. However, Price et al. [9] found no significant between-group difference in HbA1c change (−0.5% vs. −0.2%; p = 0.74) at 12 weeks, although TIR improved in the RT-CGM group. Notably, the benefits were not sustained at longer follow-up (nine months), suggesting the need for ongoing CGM use to maintain glycemic improvements.

Secondary outcomes, particularly TIR (70-180 mg/dL), were consistently improved with RT-CGM. Martens et al. [15] reported a 15% increase in TIR (59% vs. 43%; p < 0.001) and a 16% reduction in time spent >250 mg/dL (p < 0.001) compared to BGM. Furler et al. [17] also observed a significant increase in TIR (+7.9%, 95% CI 2.3-13.5; p = 0.006) at 12 months with intermittent professional CGM use. Wada et al. [11] highlighted additional benefits of FGM, including reduced glycemic variability and improved treatment satisfaction, despite a modest HbA1c reduction (−0.29%, p = 0.022).

Subgroup and Sensitivity Analyses

Subgroup analyses revealed differential efficacy based on intervention frequency and patient characteristics. Moon et al. [10] demonstrated that two intermittent RT-CGM sessions (three months apart) led to sustained HbA1c reduction (−0.68%, p = 0.018) at six months, whereas a single session did not. Haak et al. [16] found no overall HbA1c difference but noted significant improvements in participants aged <65 years, alongside reduced hypoglycemia and higher treatment satisfaction. Ajjan et al. [12] reported that multiple sensor wears (Group C: four wears) significantly reduced HbA1c (−0.48%, p = 0.0041) compared to SMBG alone, without increasing hypoglycemia.

Safety and Patient-Reported Outcomes

RT-CGM was generally safe, with no studies reporting severe device-related adverse events. Hypoglycemia outcomes were heterogeneous; Yeoh et al. [13] noted a non-significant increase in hypoglycemia exposure with retrospective CGM, while Ajjan et al. [12] observed no increase in hypoglycemia despite improved euglycemia. Treatment satisfaction scores were higher in RT-CGM groups across multiple studies [11,14,16], as detailed in Table 2.

**Table 2:** Glycemic outcomes from the included studies RT-CGM: real-time continuous glucose monitoring, SMBG: self-monitoring of blood glucose, HbA1c; hemoglobin A1c (glycated hemoglobin), FGM; flash glucose monitoring, CGM: continuous glucose monitoring, TG1: treatment group 1, TG2: treatment group 2, SD: standard deviation, BGM: blood glucose monitoring, DTSQ: Diabetes Treatment Satisfaction Questionnaire, PAID: problem areas in diabetes, NR: not reported, DM: diabetes mellitus, CI: confidence interval, mmol/mol: millimoles per mole, mmol/L: millimoles per liter, mg/dL: milligrams per deciliter, h/day: hours per day

Author (year)	Intervention group (RT-CGM)	Comparator group	Primary outcome measure	Baseline value (intervention / comparator)	Follow-up value (intervention / comparator)	Mean difference (95% CI) / p-value	Secondary outcomes
Price et al. [9] (2021)	RT-CGM (n = 45); episodic use at weeks 0, 4, and 8	SMBG (n = 23)	HbA1c change at week 12	8.4% (0.8) / 8.3% (1.2)	-0.5% (1.3) / -0.2% (1.1)	Between-group difference p = 0.74	Time in range (70–180 mg/dL) increased from 56.3% (24.5) to 63.1% (25.5) in the RT-CGM group vs. decreased from 68.4% (21.5) to 55.1% (30.3) in the SMBG group; % achieving HbA1c <7.5% at week 12: 34.1% RT-CGM vs. 17.4% SMBG (p = 0.12); HbA1c reduction not sustained at month 9
Moon et al. [10] (2023)	Treatment group 1 (one session of RT-CGM); treatment group 2 (two sessions of RT-CGM with a three-month interval)	Control group (blinded CGM only + education)	Change in HbA1c at six months	Overall baseline HbA1c: 8.2% ± 0.5% (specific group-wise baseline not reported)	At three months: TG1: –0.60% vs. control (p = 0.044); TG2: –0.64% vs. control (p = 0.014). At six months: TG2: –0.68% vs. control (p = 0.018); TG1 not significant at six months.	TG1 at 3m: –0.60%, p = 0.044; TG2 at 3m: –0.64%, p = 0.014; TG2 at 6m: –0.68%, p = 0.018	HbA1c reduction was significant only in those performing SMBG ≥1.5 times/day at three and six months; no significant improvement in SMBG <1.5/day.
Wada et al. [11] (2020)	FGM, n = 49	Self-monitoring of blood glucose (SMBG), n = 51	Change in HbA1c (%)	7.83% / 7.84%	At 12 weeks: 7.40% / 7.54%; at 24 weeks: 7.37% / 7.67%	At 24 weeks: between-group difference −0.29% (−3.2 mmol/mol), p = 0.022	Significant improvement in the Diabetes Treatment Satisfaction Questionnaire score; decreased mean glucose levels, SD of glucose, mean amplitude of glycemic excursions, and time in hyperglycemia in the FGM group compared to SMBG
Ajjan et al. [12] (2019)	Group C: SMBG + four Libre Pro sensor wears	Group A: SMBG only	Time in range (3.9–10 mmol/L) within group C	15.0 ± 5.0 h/day (intervention only reported)	14.1 ± 4.7 h/day (intervention only reported)	p = 0.1589 (within-group C comparison)	HbA1c reduced within group C by 4.9 ± 8.8 mmol/mol (0.44% ± 0.81%; p = 0.0003); HbA1c lower in group C vs A by 5.4 ± 1.79 mmol/mol (0.48% ± 0.16%; p = 0.0041); no increase in hypoglycemia (p = 0.1795); improved treatment satisfaction (p = 0.0225)
Yeoh et al. [13] (2018)	Retrospective CGM-guided anti-diabetic therapy	Self-monitoring of blood glucose (SMBG)	HbA1c (%)	9.8 ± 1.2 / 9.9 ± 1.3	8.8 ± 1.8 / 9.1 ± 1.1	No significant difference between groups (p = 0.869); within-group p-value CGM: 0.009; SMBG: 0.007	CGM group: % time in hyperglycemia (>10 mmol/L) reduced from 65.4 ± 22.4% to 54.6 ± 23.6% (p = 0.033); % time in hypoglycemia increased non-significantly from 1.2 ± 2.2% to 4.0 ± 7.0% (p = 0.176)
Sato et al. [14] (2016)	Treatment guidance based on CGM data	Advice based on blood glucose and HbA1c levels	HbA1c (%)	8.2 ± 1.2 / 8.2 ± 0.9	Significant difference in change from baseline	Not significant	Diabetes Treatment Satisfaction Questionnaire score
Martens et al. [15] (2021)	CGM (n = 116)	BGM (n = 59)	HbA1c at eight months	9.1% / 9.0%	8.0% / 8.4%	-0.4% (95% CI, -0.8% to -0.1%); p = 0.02	- Mean % time in target range (70–180 mg/dL): 59% vs. 43% (difference +15%, 95% CI 8%-23%, p < 0.001) - Mean % time >250 mg/dL: 11% vs. 27% (difference -16%, 95% CI -21% to -11%, p < 0.001) - Mean glucose: 179 mg/dL vs. 206 mg/dL (difference -26 mg/dL, 95% CI -41 to -12, p < 0.001)
Haak et al. [16] (2017)	Flash glucose-sensing technology (RT-CGM)	SMBG (self-monitoring of blood glucose)	HbA1c at six months	Not reported	Change: −3.1 ± 0.75 mmol/mol (−0.29 ± 0.07%) / −3.4 ± 1.04 mmol/mol (−0.31 ± 0.09%)	No significant difference; p = 0.8222	Time in hypoglycemia <3.9 mmol/L reduced by 0.47 ± 0.13 h/day (p = 0.0006); <3.1 mmol/L reduced by 0.22 ± 0.07 h/day (p = 0.0014); SMBG frequency reduced from 3.8 ± 1.4 to 0.3 ± 0.7 tests/day in intervention; Treatment satisfaction higher (DTSQ 13.1 ± 0.50 vs. 9.0 ± 0.72; p < 0.0001)
Furler et al. [17] (2020)	Professional-mode flash glucose monitoring (Freestyle Libre Pro) at three-month intervals for 12 months	Usual clinical care	Mean HbA1c at 12 months	Not reported	8.2% (95% CI 8.0–8.4) Intervention vs. 8.5% (8.3–8.7) Comparator	−0.3% (95% CI −0.5 to 0.01); p = 0.059	- Mean HbA1c at six months: −0.5% (95% CI −0.8 to −0.3); p = 0.0001- % Time in target glucose range (4–10 mmol/L) at 12 months: +7.9% (95% CI 2.3–13.5); p = 0.0060- Diabetes-specific distress (PAID score): no significant difference (−0.7, 95% CI −3.3 to 1.9; p = 0.61)
Beck et al. [18] (2017)	RT-CGM (n = 79)	Usual care (n = 79)	HbA1c reduction at 24 weeks	8.5% (both groups)	7.7% / 8.0%	−0.3% (95% CI, −0.5% to 0.0%); p = 0.022	CGM-measured hypoglycemia (no meaningful difference); quality-of-life outcomes (no meaningful difference)
Ajjan et al. [19] (2016)	RT-CGM with ambulatory glucose profile (type 2 DM, n = 28)	Capillary glucose testing	HbA1c (mmol/mol)	77 ± 15 / NR	67 ± 13 / NR	p = 0.0002	↑ time in euglycemia by 1.4 ± 3.5 h/day (p = 0.0427); no ↑ hypoglycemia

Limitations and Heterogeneity

The studies exhibited clinical and methodological heterogeneity, including variations in CGM device type (e.g., professional vs. personal use), intervention duration, and patient populations (e.g., insulin vs. non-insulin-treated). Some trials, such as Sato et al. [14], reported no significant HbA1c differences, possibly due to shorter CGM exposure or lack of real-time feedback.

RT-CGM shows promise in improving glycemic control, particularly TIR and HbA1c, in adults with T2D, although its efficacy depends on the frequency of use and patient subgroup. Sustained benefits require ongoing CGM adherence, and further research is needed to optimize implementation strategies.

Risk-of-Bias Findings

**Figure 2:**
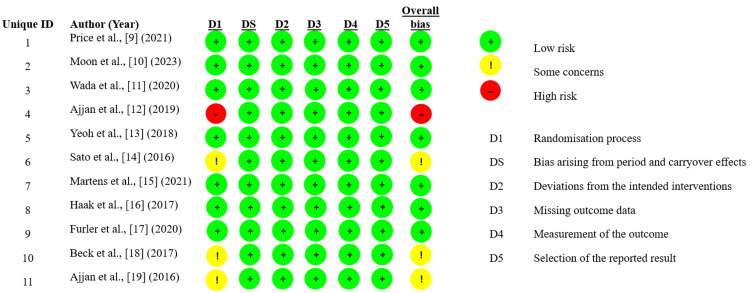
Risk-of-bias assessment using the Risk of Bias (RoB) 2 tool

The risk-of-bias assessment using the Cochrane RoB 2 tool revealed that the majority of the included studies were judged to have a low risk of bias across all domains. Specifically, Price et al. [9], Moon et al. [10], Wada et al. [11], Yeoh et al. [13], Martens et al. [15], Haak et al. [16], and Furler et al. [17] were assessed as low risk in all domains, indicating robust methodological quality. Sato et al. [14], Beck et al. [18], and Ajjan et al. [19] had some concerns in at least one domain, primarily related to the randomization process or selection of reported results, suggesting minor methodological limitations. Notably, Ajjan et al. [12] were rated as having a high risk of bias in the randomization process and overall, which may affect the credibility of their findings (Figure 2).

Discussion

The findings of this systematic review demonstrate that RT-CGM offers clinically meaningful benefits for glycemic management in adults with T2D, although its efficacy varies based on intervention protocols, patient subgroups, and outcome measures. The included studies collectively suggest that RT-CGM can significantly improve TIR and reduce HbA1c, particularly when used consistently or with structured interventions. For instance, Martens et al. [15] reported a 15% increase in TIR (70-180 mg/dL) and a 0.4% reduction in HbA1c compared to traditional BGM, underscoring the potential of RT-CGM to enhance glycemic stability. Similarly, Beck et al. [18] observed a modest but statistically significant HbA1c reduction (−0.3%) with RT-CGM, aligning with evidence that continuous glucose data facilitate more precise therapy adjustments. However, not all studies reported uniform benefits; Price et al. [9] found no significant between-group difference in HbA1c at 12 weeks, although TIR improved, suggesting that glycemic outcomes may depend on the frequency and duration of RT-CGM use. This heterogeneity mirrors findings from broader literature, where intermittent CGM use often yields smaller HbA1c reductions compared to continuous use, as noted by Jackson et al. [4].

A key strength of RT-CGM lies in its ability to mitigate glycemic variability, a factor increasingly recognized as an independent risk factor for diabetes complications. Wada et al. [11] highlighted that FGM reduced the standard deviation of glucose and time in hyperglycemia, corroborating studies by Rigon et al. [5], which emphasized CGM’s role in minimizing glucose excursions. Notably, secondary outcomes such as treatment satisfaction were consistently higher in RT-CGM groups across multiple trials, including Haak et al. [16] and Ajjan et al. [12]. This aligns with qualitative research indicating that patients value real-time alerts and trend data for empowering self-management, as described by Préau et al. [6]. However, the lack of sustained HbA1c benefits in some studies, such as Price et al. [9], where improvements waned by nine months, raises questions about long-term adherence and the need for ongoing CGM integration into care routines.

Subgroup analyses revealed nuanced insights into RT-CGM’s efficacy. Moon et al. [10] demonstrated that two intermittent RT-CGM sessions led to sustained HbA1c reductions (−0.68%) at six months, whereas a single session did not, suggesting that periodic “doses” of CGM data may be necessary for lasting effects. This finding resonates with clinical guidelines recommending episodic CGM for non-insulin-treated T2D patients, as outlined by the American Diabetes Association [20]. Similarly, Haak et al. [16] reported age-specific benefits, with significant HbA1c reductions in participants under 65 years, possibly reflecting greater engagement with technology or more aggressive therapy adjustments. Such variability underscores the importance of personalized CGM strategies tailored to patient demographics and treatment regimens.

Safety and hypoglycemia outcomes were generally favorable, with no studies reporting severe device-related adverse events. Ajjan et al. [12] observed no increase in hypoglycemia despite improved euglycemia, a critical advantage given the risks associated with intensive glucose-lowering therapies. Conversely, Yeoh et al. [13] noted a non-significant rise in hypoglycemia with retrospective CGM, highlighting that hypoglycemia risk may depend on how CGM data are acted upon. These findings align with broader evidence that RT-CGM’s real-time alerts reduce hypoglycemia in insulin-treated patients, as demonstrated in trials by Martens et al. [15], but may have limited impact in populations where hypoglycemia is less prevalent, such as those on non-insulin therapies.

The methodological heterogeneity across studies, including variations in CGM devices (e.g., professional vs. personal use) and comparator groups (e.g., SMBG frequency), complicates direct comparisons. For example, Sato et al. [14] used retrospective CGM without real-time feedback and found no HbA1c benefit, whereas Furler et al. [17] employed intermittent professional CGM with clinician review and reported improved TIR. This discrepancy underscores the importance of real-time data visibility and clinician engagement in driving glycemic improvements, as emphasized in a meta-analysis by Galicia-Garcia et al. [1]. In addition, the predominance of open-label designs in included trials [11,16] introduces performance bias, although the objective nature of HbA1c measurements mitigates detection bias.

Compared to existing literature, this review’s findings reinforce the growing consensus that RT-CGM is superior to SMBG for glycemic control in T2D, particularly for insulin-treated patients. A 2022 meta-analysis by Pleus et al. [3] reported pooled HbA1c reductions of 0.3-0.5% with RT-CGM, consistent with our results. However, our review adds nuance by identifying subgroups (e.g., younger patients, those with structured CGM protocols) who derive the greatest benefit, a gap noted in prior syntheses. The contrast between our findings and older studies, such as those focusing on blinded CGM [21], highlights the transformative impact of real-time feedback on patient and clinician behavior.

Limitations

Despite its strengths, this review has several limitations. First, the predominance of open-label trials risks performance bias, although objective outcomes (e.g., HbA1c) lessen this concern. Second, heterogeneity in intervention protocols and follow-up durations precluded meta-analysis, limiting quantitative synthesis. Third, most studies had short follow-ups (<12 months), obscuring long-term efficacy and sustainability. Finally, the exclusion of non-English studies and those involving type 1 diabetes or pregnant individuals may restrict generalizability.

## Conclusions

RT-CGM is a valuable tool for improving glycemic outcomes in adults with T2D, particularly for enhancing TIR and reducing HbA1c in insulin-treated and engaged populations. Its benefits are most pronounced with consistent use or structured intermittent protocols, supported by clinician involvement. Future research should prioritize long-term trials, standardized outcome reporting, and cost-effectiveness analyses to guide broader implementation. Clinically, these findings advocate for individualized RT-CGM adoption, emphasizing patient education and interdisciplinary care to maximize its potential.
